# *Streptococcus bovis*-bacteremia: subspecies distribution and association with colorectal cancer: a retrospective cohort study

**DOI:** 10.1017/S0950268821002533

**Published:** 2021-11-26

**Authors:** Jonas Öberg, Magnus Rasmussen, Pamela Buchwald, Bo Nilson, Malin Inghammar

**Affiliations:** 1 Department of Clinical Sciences Lund, Section for Infection Medicine, Lund University, Lund, Sweden; 2 Department of Infectious Diseases, Helsingborg Hospital, Helsingborg, Sweden; 3 Department of Surgery, Skåne University Hospital Malmö, Malmö, Sweden; 4 Department of Clinical Sciences Malmö, Lund University, Lund, Sweden; 5 Department of Laboratory Medicine Lund, Section of Medical Microbiology, Lund University, Lund, Sweden; 6 Department of Clinical Microbiology, Infection Control and Prevention, Office for Medical Services, Region Skåne, Lund, Sweden

**Keywords:** Colorectal cancer, *S. bovis/equinus* complex (SBSEC), *S. gallolyticus* subsp. *Gallolyticus*, *S. gallolyticus* subsp. *pasteurianus*, *Streptococcus bovis*

## Abstract

This study aimed to describe the incidence of *Streptococcus bovis/Streptococcus equinus* complex (SBSEC) bacteremia, distribution of the SBSEC subspecies, and their respective association with colorectal cancer (CRC). A population-based retrospective cohort study of all episodes of SBSEC-bacteremia from 2003 to 2018 in Skåne Region, Sweden. Subspecies was determined by whole-genome sequencing. Medical charts were reviewed. The association between subspecies and CRC were analysed using logistic regression. In total 266 episodes of SBSEC-bacteremia were identified and the average annual incidence was 2.0 per 100 000 inhabitants. Of the 236 isolates available for typing, the most common subspecies was *S. gallolyticus* subsp. *pasteurianus* 88/236 (37%) followed by *S. gallolyticus* subsp. *gallolyticus* 58/236 (25%). In order to determine the risk of cancer following bacteremia, an incidence cohort of 174 episodes without a prior diagnosis of CRC or metastasised cancer was followed for 560 person-years. CRC was found in 13/174 (7%), of which 9 (69%) had *S. gallolyticus* subsp. *gallolyticus-*bacteremia. In contrast to other European studies, *S. gallolyticus* subsp. *pasteurianus* was the most common cause of SBSEC-bacteremia. CRC diagnosis after bacteremia was strongly associated with *S. gallolyticus* subsp. *gallolyticus*-bacteremia. Identification of SBSEC subspecies can guide clinical decision-making regarding CRC work-up following bacteremia.

## Introduction

Bacteria and viruses may have a role in carcinogenesis, the most well-known examples are the associations between *Human papillomavirus* and cervical cancer, and *Helicobacter pylori* and gastric cancer. Colorectal cancer (CRC) is the third most common cancer in Sweden and worldwide [1]. The incidence in Sweden is 62 per 100 000 population and increasing [2]. Despite this, the role of the gut microbiota in CRC carcinogenesis is yet sparsely studied.

*S. bovis-S. equinus* complex (SBSEC) is a group of bacteria found in the gastrointestinal canal, including *S. gallolyticus* subsp. *gallolyticus (Sg gallolyticus)*, *S. gallolyticus* subsp. *pasteurianus (Sg pasteurianus)*, *S. gallolyticus* subsp. *macedonicus*, *S. lutetiensis* (also known as *S. infantarius* subsp. *coli)*, *S. infantarius* subsp. *infantarius (Si infantarius)*, *S. equinus* and *S. alactolyticus* [3–5]. In 1979, Klein *et al*. described the nowadays well-known association between bacteremia with SBSEC and CRC [6]. In recent years, studies have indicated that the association may be largely dependent on subspecies, with the strongest association reported for *Sg gallolyticus* [5, 7–9]. However, most studies have been single-centre, often with a limited or skewed subspecies distribution depending on study population and region [8, 10–12]. Additionally, some studies have suggested a possible association with other gastrointestinal malignancies [11–13].

Methods used for subspecies identification of SBSEC in clinical routine have many pitfalls and classifications have changed over time [14–17]. Whole-genome sequencing (WGS) provides more accurate identification and has not previously been used in epidemiological studies of SBSEC.

The purpose of this study was to determine the incidence of SBSEC bacteremia and subspecies distribution using WGS and to investigate the risk of CRC and other gastrointestinal cancer for each subspecies following bacteremia.

## Methods

### Settings and study design

A retrospective population-based study was conducted of the incidence of SBSEC-bacteremia, subspecies distribution and association with CRC and other gastrointestinal cancer in Skåne Region, from January 2003 to December 2018. Skåne region has 1.4 million inhabitants as of 31 December 2018, of which 91% are residing in urban areas (localities as defined by Statistics Sweden) [18]. Skåne is one of the leading regions in agricultural production, both crops and livestock, in Sweden.

Data of all episodes of positive blood cultures with SBSEC were obtained from the laboratory databases of Clinical Microbiology, Region Skåne, Lund, Sweden, the only laboratory serving all ten hospitals in Skåne Region. MALDI-TOF MS, Microflex with the MALDI Biotyper software (Bruker), analysis of positive blood cultures was introduced in 2012, before that isolates were identified by biochemical methods and/or 16S rRNA gene sequencing. Microbial and clinical records from databases covering all hospitals of Skåne Region were reviewed retrospectively. A unique, lifelong ten-digit personal identity number assigned to all persons living in Sweden, provides the possibility of cross-referencing information in health databases. Only the first positive blood culture per hospital admission was considered an episode.

### Microbiology

SBSEC isolates from blood cultures stored in −70 °C at Clinical Microbiology, and reference type strain (Table S1) for each SBSEC subspecies were precultured on blood agar plates in 5% CO_2_ at 37 °C for 24–120 h and grown in Brain heart infusion broth at 37 °C, 120 shakes/min, for 12–48 h. DNA was purified using the DNeasy Blood & tissue kit (QIAGEN, Inc.) according to the manufacturer's description.

Short read WGS was performed at The Center for Translational Genomics, Lund University. DNA samples were processed using Nextera XT DNA Library Prep (Illumina, Inc.), and sequenced using NextSeq 500 or NovaSeq 6000 instrument (Ilumnia, Inc.) with NovaSeq 6000 SP Reagent Kit (Ilumnia, Inc.) and Phix Control (Ilumnia, Inc.). Cycle parameters used were: read 1: 151, index read 1: 8, index read 2: 8, read 2: 151. Kraken 2 with Bracken was used for confirmation of SBSEC-species and inter-species contamination control [19, 20]. Intra-species contamination was assessed by quantification of polymorphic loci. SNP analysis was performed using Snippy [21]. A neighbour-joining phylogenetic tree using Kimura-2-parameter was constructed using SeaView [22]. Subspecies was confirmed by clusters forming around reference type strains for each SBSEC subspecies (Figure S1).

A new episode in the same patient was presumed to be caused by the same subspecies if the isolate was unavailable from new episode.

### Data collection

Medical charts were reviewed and data were gathered according to a predefined protocol. Data collection included age, sex, comorbidities and focus of infection. Examination by colonoscopy or computed tomography colonography within 12 months, macroscopic findings and biopsy results och cancer, and tubular-, villous-, tubulovillous- and serrated adenoma were registered. Diagnoses of cancer prior to and after positive blood culture, i.e. prevalent and incident cancer, were collected from the regional pathological database, covering the total population and all caregivers in Skåne Region. Cancer diagnosed during the hospital stay but before positive SBSEC blood culture was registered as prevalent cancer. Other gastrointestinal cancer was defined as oesophagus-, gastric-, small bowel-, liver-, biliary-, gallbladder-, or pancreatic cancer. Individuals were followed until the date of death, emigration or 15 March 2021. The Charlson index was used for the categorisation of comorbidities [23]. The focus of infection was defined by the discretion of the physicians.

### Statistical methods

Chi-squared and Kruskal Wallis' tests were used to assess differences in the distribution of background factors or incident CRC between episodes with different SBSEC subspecies.

Annual incidence rates for SBSEC-bacteremia were calculated from 2012, the introduction of MALDI-TOF MS testing of bacterial isolates from blood cultures, by dividing the number of episodes per year by the population denominator in Skåne Region 31 December each year [18]. Individuals with missing data were included in the calculation of incidence rates.

In order to determine the risk of CRC and other gastrointestinal cancer following SBSEC-bacteremia, analyses were restricted to individuals without a prior diagnosis of CRC or metastasised cancer at the time of bacteremia – the incidence cohort. The association between subspecies and CRC, adjusted for potential confounders selected *a priori*, was analysed using logistic regression [1, 2, 9]. The covariates were investigated separately and in a full model including SBSEC-subspecies (*Sg gallolyticus versus* other SBSEC-bacteremia), age groups (≤70, 71–80 and ≥81 years), sex, and if a focus of infection was determined. The area under the receiving operator characteristic (ROC) curve was used to assess the capacity to predict CRC.

Numbers needed to screen (NNS) were calculated for the incidence cohort for diagnosis of CRC within 12 months, for all SBSEC-bacteremia and *Sg gallolyticus*-bacteremia respectively; overall and for the subgroup with an unknown focus of infection. Standardised incidence ratio of CRC diagnosis within 12 months was analysed for *Sg gallolyticus* and other SBSEC-bacteremia, with the CRC incidence in Skåne Region as a reference, stratified by calendar year, 5-year interval age group and sex [24]. In this calculation, only the primary episode per individual per subspecies for the whole cohort was included to avoid bias.

Statistical analysis was performed using Stata/SE version 16 (StataCorp. College Station, TX).

### Ethics

Data were anonymised before analysis. The study was approved by the regional Ethics Committee in Lund (Dnr 2018/429).

## Results

### Incidence, subspecies distribution and study cohort

**Fig. 1.**
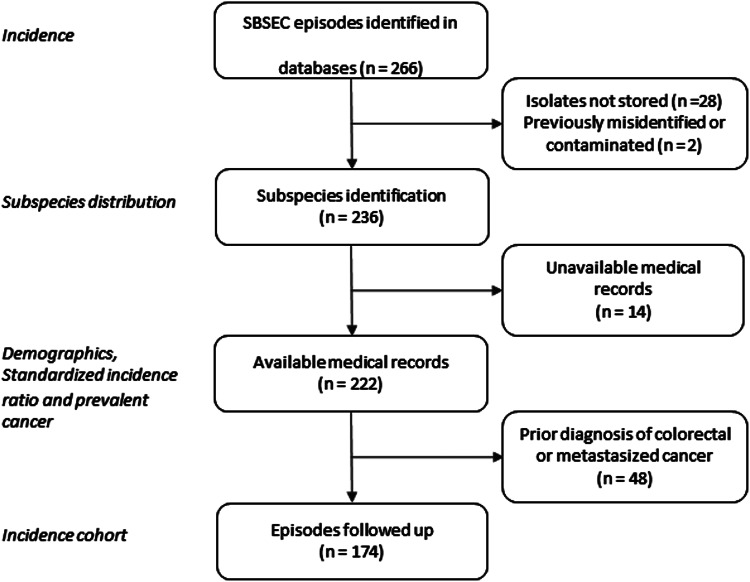
Flow chart of selection of SBSEC bacteremia episodes.

In total, 266 episodes of SBSEC-bacteremia in 252 individuals were identified, corresponding to an average annual incidence rate of 2.03 (range 1.35 to 2.94) per 100 000 inhabitants (Fig. 1, Table S2).

**Fig. 2.**
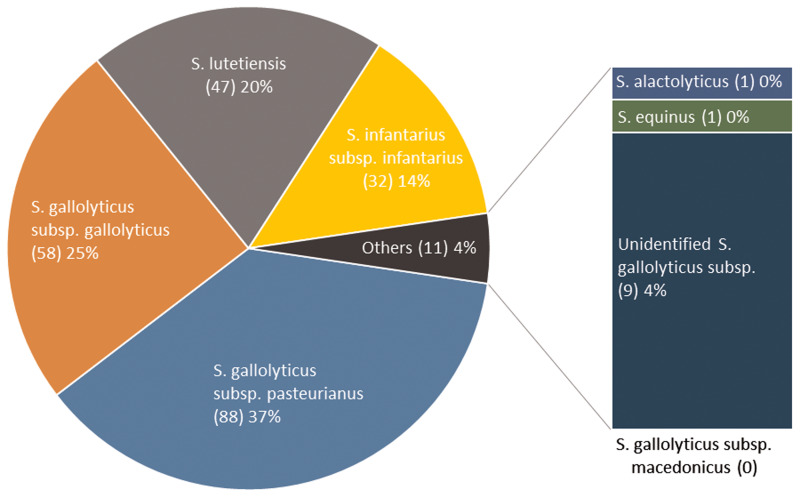
SBSEC subspecies distribution.

Two hundred thirty-six isolates were available for WGS and identified as *Sg pasteurianus* 88 (37%), *Sg gallolyticus* 58 (25%), *S. lutetiensis* 47 (20%), *Si infantarius* 32 (14%) and other subspecies 11 (4%) (Fig. 2). Nine episodes formed a separate cluster among the *S. gallolyticus* subspecies and could not be assigned a definite subspecies. Medical records were unavailable in 14 episodes, leaving 222 episodes in 212 individuals available for analysis. Demographics are presented in Table 1.

**Table 1.** Background characteristics^a^ according to groups of subspecies of SBSEC

	*S. gallolyticus* subsp. *pasteurianus* (*n* = 83)	*S. gallolyticus* subsp. *gallolyticus* (*n* = 53)	*S. lutetiensis* (*n* = 44)	*S. infantarius* subsp. *infantarius* (*n* = 31)	Other subspecies^b^ (*n* = 11)	*P*
Age, years	76 (66–86)	78 (73–85)	73 (63–81)	81 (74–86)	80 (72–82)	0.11
Male	43 (52)	30 (57)	28 (64)	22 (71)	11 (100)	0.02
Charlson comorbidity index score: 0–2	42 (51)	27 (51)	16 (36)	15 (48)	8 (73)	0.03
3–4	28 (34)	15 (28)	10 (23)	6 (19)	0 (0)	
≥5	13 (16)	11 (21)	18 (41)	10 (32)	3 (27)	
Unknown focus of infection^c^	32 (39)	23 (44)	16 (36)	16 (52)	7 (64)	0.36
Prevalent cancer, diagnosed before known bacteremia						
Colorectal adenoma/polyps	13 (16)	11 (21)	8 (18)	9 (29)	0 (0)	0.25
Colorectal cancer, all	12 (14)	4 (8)	6 (14)	3 (10)	0 (0)	0.51
Colorectal cancer, diagnosed within 3 months^d^	4 (5)	1 (2)	1 (2)	0 (0)	0 (0)	0.60
Colorectal cancer, metastasised	5 (6)	0 (0)	4 (9)	0 (0)	0 (0)	0.10
Pancreatic cancer	0 (0)	0 (0)	3 (7)	2 (6)	0 (0)	0.04
Gallbladder-/bile duct cancer	2 (2)	1 (2)	1 (2)	1 (3)	0 (0)	0.98
Oesophagus-, gastric-, small bowel- and liver cancer	3 (4)	0 (0)	1 (2)	2 (6)	0 (0)	0.44
Non gastrointestinal cancer	23 (28)	14 (26)	11 (25)	12 (39)	2 (18)	0.63
Cancer, in total^e^	36 (43)	18 (34)	21 (48)	18 (58)	2 (18)	0.10
Metastasised cancer, in total^e^	9 (11)	3 (6)	13 (30)	5 (16)	2 (18)	0.01

a Categorical variables are presented as *n* (%), ordinal variables are presented as median (IQR).

b *S. alactolyticus*, *S. equinus*, unidentified *S. gallolyticus* subspecies.

c 1 episode of *S. gallolyticus* subsp *gallolyticus* with missing data not included.

d 1 episode of *S. gallolyticus* subsp *pasteurianus* and 1 of *S. gallolyticus* subsp *gallolyticus* were diagnosed during the hospital stay but before SBSEC bacteremia was diagnosed.

e All cancer, in total.

### Bacteremia with *S. bovis/S. equinus* complex and cancer

#### Prevalent colorectal- and gastrointestinal cancer

Prevalent CRC, diagnosed prior to having a positive SBSEC blood culture were noted in 25/222 (11%, 22 individuals) episodes. Nine of these were metastasised, of which five had bacteremia with *Sg pasteurianus* and four with *S. lutetiensis*. There was no significant difference in the frequency of prevalent CRC, colon polyps or adenoma between the different subspecies (Table 1). Pancreatic cancer was more frequent in the *S. lutetiensis*- and *Si infantarius*-groups (*P* = 0.04).

#### Incidence of colorectal- and gastrointestinal cancer

There were 174 episodes in 168 individuals without preexisting CRC or other metastasised cancer at the time of SBSEC-bacteremia, the incidence cohort, these were followed for 560 person-years in total, with a median follow-up time per individual of 2.9 years (IQR 0.4–4.6) (Table 2). There was no loss of follow up. Of these, 72 (41%) patients underwent colonoscopy or computed tomography colonography within 12 months of bacteremia.

**Table 2.** Incident cancer diagnosed after bacteremia^a^^.^^b^

	*S. gallolyticus* subsp. *pasteurianus* (*n* = 67)	*S. gallolyticus* subsp. *gallolyticus* (*n* = 46)	*S. lutetiensis* (*n* = 29)	*S. infantarius* subsp. *infantarius* (*n* = 23)	Other subspecies^c^ (*n* = 9)	*P*
Colonoscopy or CT colonography	24 (36)	29 (63)	6 (21)	8 (35)	5 (56)	<0.01
Cancer diagnosis within 12 months						
Colorectal cancer	2 (3)	9 (20)	0 (0)	1 (4)	0 (0)	<0.01
Colorectal neoplasia^d^	6 (9)	23 (50)	6 (21)	8 (35)	4 (44)	<0.001
Other gastrointestinal cancer^e^	0 (0)	0 (0)	0 (0)	0 (0)	0 (0)	NA
Other cancer	0 (0)	0 (0)	5 (17)	0 (0)	2 (22)	<0.001
Cancer diagnosis in total during the follow-up period						
Follow-up time, years	3.3 (0.3–5.8)	3.0 (1.0–5.3)	2.4 (0.5–3.9)	1.5 (0.2–3.4)	3.4 (0.1–4.8)	0.39
Colorectal cancer	3 (4)	9 (20)	0 (0)	1 (4)	0 (0)	<0.01
Other gastrointestinal cancer^e^	0 (0)	0 (0)	1 (3)	0 (0)	0 (0)	0.28

a Calculated among patients without a prior diagnosis of CRC or metastasised cancer at the time of bacteremia.

b Categorical variables are presented as *n* (%), ordinal variables are presented as median (IQR).

c *S. alactolyticus*, *S. equinus*, unidentified *S. gallolyticus* subspecies.

d Colorectal cancer, adenomas or polyps.

e Oesophagus-, gastric-, small bowel-, liver-, biliary-, gallbladder-, and pancreatic cancer.

During follow up, 13/174 (7%, 13 individuals) were diagnosed with CRC. The majority (12/13) within 12 months, i.e. 12/72 (17%) of those who underwent colorectal work-up. Only one case of CRC was diagnosed during the remaining follow up period, 63 months after bacteremia. This individual was not examined by colonoscopy or computed tomography colonography within 12 months of bacteremia, and the focus of infection was considered unknown. The association with CRC was most pronounced with *Sg gallolyticus,* 9/46 (20%) were diagnosed with CRC as compared to 0–4% among the episodes with other subspecies (*P* < 0.01, Table 2). Details on colonoscopy findings are available in Table S3. The proportion of episodes diagnosed with CRC within 12 months were 8/74 (11%) for those with an unknown focus of infection, and 3/99 (3%) for those with a diagnosed focus. The corresponding numbers for *Sg gallolyticus* were 7/20 (35%) and 1/25 (4%), and for other SBSEC bacteremia 1/54 (2%) and 2/74 (3%). Of the three episodes with identified focus, two were infective endocarditis and one erysipelas with concurrent growth of *Staphylococcus aureus* in blood cultures. None of the episodes diagnosed with CRC had an identified intra-abdominal focus of infection (49/173, 28%). Data on the focus of infection were unavailable in one episode with *Sg gallolyticus*, later diagnosed with CRC.

NNS for diagnosis of CRC within 12 months for all episodes of SBSEC-bacteremia in the incident cohort was 15, and 9 in the subgroup with an unknown focus of infection. In episodes with *Sg gallolyticus*, NNS was 5 and 3 respectively. Adjusted odds ratios (OR) for diagnosis of CRC was 9.6 [95% confidence interval (95% CI) 2.3–40.6] after *Sg gallolyticus*-bacteremia compared to other SBSEC-bacteremia (Table 3). The area under the ROC curve for the multivariable model was 0.80. More data on subspecies and CRC are shown in Supplements.

**Table 3.** Variables associated with a colorectal cancer diagnosis within 12 months^a^^,^^b^

	CRC diagnosis (*n* = 12)	No CRC diagnosis (*n* = 162)	Univariate analysis		Multivariable analysis	
			*P*^c^	OR (95% CI)	OR (95% CI)	*P*^d^
Sex						
Female (73, 42%)	3 (25)	70 (43)	0.20	ref	ref	0.18
Male (101, 58%)	9 (75)	92 (57)		2.28 (0.60–8.74)	2.72 (0.60–12.21)	
Age, years						
≤70 (47, 27%)	1 (8)	46 (28)	0.23	ref	ref	0.40
71–80 (53, 30%)	4 (33)	49 (30)		3.76 (0.40–34.85)	1.70 (0.15–19.45)	
≥81 (74, 43%)	7 (58)	67 (41)		4.81 (0.57–40.39)	3.38 (0.37–30.94)	
Species						
Other subspecies^e^ (128, 74%)	3 (25)	125 (77)	<0.001	ref	ref	<0.01
*S. gallolyticus* subsp. *gallolyticus* (46, 26%)	9 (75)	37 (23)		10.14 (2.61–39.38)	9.61 (2.27–40.61)	
Focus of infection identified^f^						
Identified (99, 57%)	3 (27)	96 (59)	0.04	ref	ref	0.07
Unknown (74, 43%)	8 (73)	66 (41)		3.88 (1.00–15.16)	3.69 (0.85–16.01)	

a Calculated among patients without a prior diagnosis of CRC or metastasised cancer at the time of bacteremia.

b Variables are presented as a number of episodes, and per cent of observations in columns in parenthesis.

c Chi-squared.

d Likelihood ratio.

e *S. gallolyticus* subsp. *pasteurianus*, *S. lutetiensis, S. infantarius* subsp. *infantarius, S. alactolyticus*, *S. equinus* and unidentified *S. gallolyticus* subspecies.

f 1 episode of *S. gallolyticus* subsp. *gallolyticus* with missing data later diagnosed with colorectal carcinoma not included in analysis.

The standardised incidence ratio of CRC diagnosis within 12 months was 59.8 (95% CI 27.3–113.3) for *Sg gallolyticus* and 7.2 (95% CI 1.5–20.9) for other SBSEC-bacteremia compared to the general population in Skåne stratified by age, sex and calendar year.

Only one case of other gastrointestinal cancer was diagnosed during follow-up, pancreatic cancer 51 months after *S. lutetiensis*-bacteremia (Table 2).

## Discussion

### Summary of main findings

In this population-based cohort study, we found that the association of SBSEC-bacteremia and CRC is mostly dependent on subspecies, with a diagnosis of CRC following *Sg gallolyticus*-bacteremia being substantially more frequent than in the general population. Most cases of CRC were diagnosed in patients with an unknown primary focus of SBSEC infection, and no patient was diagnosed with CRC after SBSEC bacteremia with intra-abdominal focus regardless of subspecies. Individuals suffering from other gastrointestinal cancer had the episodes of bacteremia after their diagnosis of cancer.

#### Comparison with other studies

The average annual incidence rate of SBSEC bacteremia was 2.0 per 100 000 inhabitants, similar to the previously reported incidence of 2.0 in Galicia, Spain [10]. *Sg pasteurianus* was the most numerous subspecies, accounting for one-third of identified isolates, and is usually predominant in studies in Asia [11]. Previous studies in Europe have reported a dominance of *Sg gallolyticus*, accounting for 36–52% of SBSEC-bacteremia, compared to 25% in the present study [8, 10]. We found *S. lutetiensis* and *Si infantarius* in 34% of bacteremia, notably higher than in most studies but similar to a study by Marmolin *et al*. (36%) in Denmark, geographically close to Skåne region [8]. These differences may be due to the method of subspecies identification, differences in microbiota, patient demographics, local recommendations on when to draw blood cultures, or other unknown epidemiological factors. Although previously diagnosed cancer was common among all subspecies, half of bacteremia episodes by *S. lutetiensis* and *Si infantarius* had previously diagnosed cancer and as many as one-third of episodes with *S. lutetiensis* had metastasised cancer.

In line with previous reports, we observed the strongest association between CRC and *Sg gallolyticus* with similar numbers as previously reported. Marmolin *et al*. reported CRC in 16% of *Sg gallolyticus* bacteremia, and Corredoira-Sanchéz *et al*. in 8% of infective endocarditis and 22% of other bacteremia [7, 8]. In another study by Corredoira using previous taxonomy, bacteremia with *S. bovis* biotype I (now *Sg* gallolyticus) was found to be the only significant risk factor for advanced adenomas or CRC following SBSEC-bacteremia (OR 9.1, 95% CI 1.8–47.8) [25]. Kwong *et al*. suggested *Sg gallolyticus* to be the only SBSEC subspecies associated with increased risk of CRC diagnosis following bacteremia compared to controls with negative blood cultures (HR 5.7, 95% CI 2.2–15.1) [9]. The reported frequencies of CRC findings vary when colonoscopy is performed due to gastrointestinal symptoms, NNS of 7–20 have been reported [26]. In the present study, only five patients with *Sg gallolyticus*-bacteremia needed to be screened, and three in individuals who also had an unknown focus of infection.

The mechanism underlying the association between CRC and SBSEC-bacteremia has yet to be explored. SBSEC-bacteremia may merely be a marker of impaired gut barrier [27]. However, recent experimental studies have implicated that *Sg gallolyticus* may have a more direct role in CRC development [28–30].

#### Limitations and strengths

Our study has some important limitations. Firstly, the routine method for species identification has changed during the study period. There was on average a threefold increase in SBSEC episodes per year since the introduction of MALDI-TOF MS in 2012, while the population in the region only increased by 19% during the study period [18]. It is likely that some SBSEC isolates may have been classified as ‘alpha-hemolytic streptococci’ previously. The substantial increase in SBSEC findings may also be due to an increased number of blood cultures drawn, or a change in patient demographics. The number of blood culture sets per 1000 patient days in Sweden was reported to be 107 in 2018 while only 67 in 2011 [31,32]. Secondly, older isolates and isolates from early relapses were stored to a lesser extent. Due to these factors, the annual incidence was calculated only for the time period after the introduction of MALDI-TOF MS in routine diagnostics, and sensitivity analysis of subspecies distribution for this period showed nearly identical subspecies distribution. We included all episodes of bacteremia for each individual, but this did not affect the number of cancer cases discovered during follow-up and thereby not the conclusions drawn. Due to the retrospective design, colonoscopy or computed tomography colonography were not systematically performed in all patients with SBSEC-bacteremia, and unevenly distributed among the subspecies. Reassuringly, only one patient was diagnosed with CRC >1 year after bacteremia, during 560 person-years of follow up, indicating that most CRC was discovered. Although the analysis of the subspecies-CRC association was adjusted for potential confounders, residual confounding from other factors such as persisting gastrointestinal symptoms and clinical findings cannot be ruled out.

The strengths of this study include the population-based design, a long follow up time in a defined region served by one microbiological- and pathological laboratory. This is the first epidemiological study of SBSEC subspecies using WGS, which minimises misclassification of SBSEC subspecies. Furthermore, this comparatively large study covers all cases of CRC and other gastrointestinal cancer, including the risk of CRC following SBSEC-bacteremia compared between the subspecies and with the rates in the general population.

#### Implications

Given the strong association with *Sg gallolyticus*, colonoscopy should always be considered following *Sg gallolyticus*-bacteremia, and in all SBSEC-bacteremia if the focus of infection is unknown. Moreover, the results indicate that colonoscopy may be redundant if there is an intra-abdominal infection source of infection.

In conclusion, our findings support that SBSEC subspecies distribution can vary, even in regions geographically close. Speculatively, variation in the regional incidence of *Sg gallolyticus*-infection may have implications for CRC incidence although this requires further research. The clinical implications for CRC work-up depend on subspecies, therefore, we recommend identification of SBSEC-bacteremia at the subspecies level.

## Data Availability

The data that support the findings of this study are available from the corresponding author upon reasonable request.

## Supplementary material

For supplementary material accompanying this paper visit http://doi.org/10.1017/S0950268821002533. click here to view supplementary material
