# Supplementary material for: Streptococcus bovis-bacteremia: subspecies distribution and association with colorectal cancer: a retrospective cohort study
Source: Epidemiol Infect. 2021 Nov 26;150:e8. doi: 10.1017/S0950268821002533 (PMC8753481; doi:10.1017/S0950268821002533)
Supplement: Supplementary file 1 [file S0950268821002533sup001.docx]

**Epidemiology and Infection**

# *Streptococcus bovis*-bacteremia: subspecies distribution and association with colorectal cancer – a retrospective cohort study

Jonas Öberg*, MD; Magnus Rasmussen, MD, Prof; Pamela Buchwald, MD, PhD; Bo Nilson, PhD; Malin Inghammar, MD, PhD.

Supplementary material

**
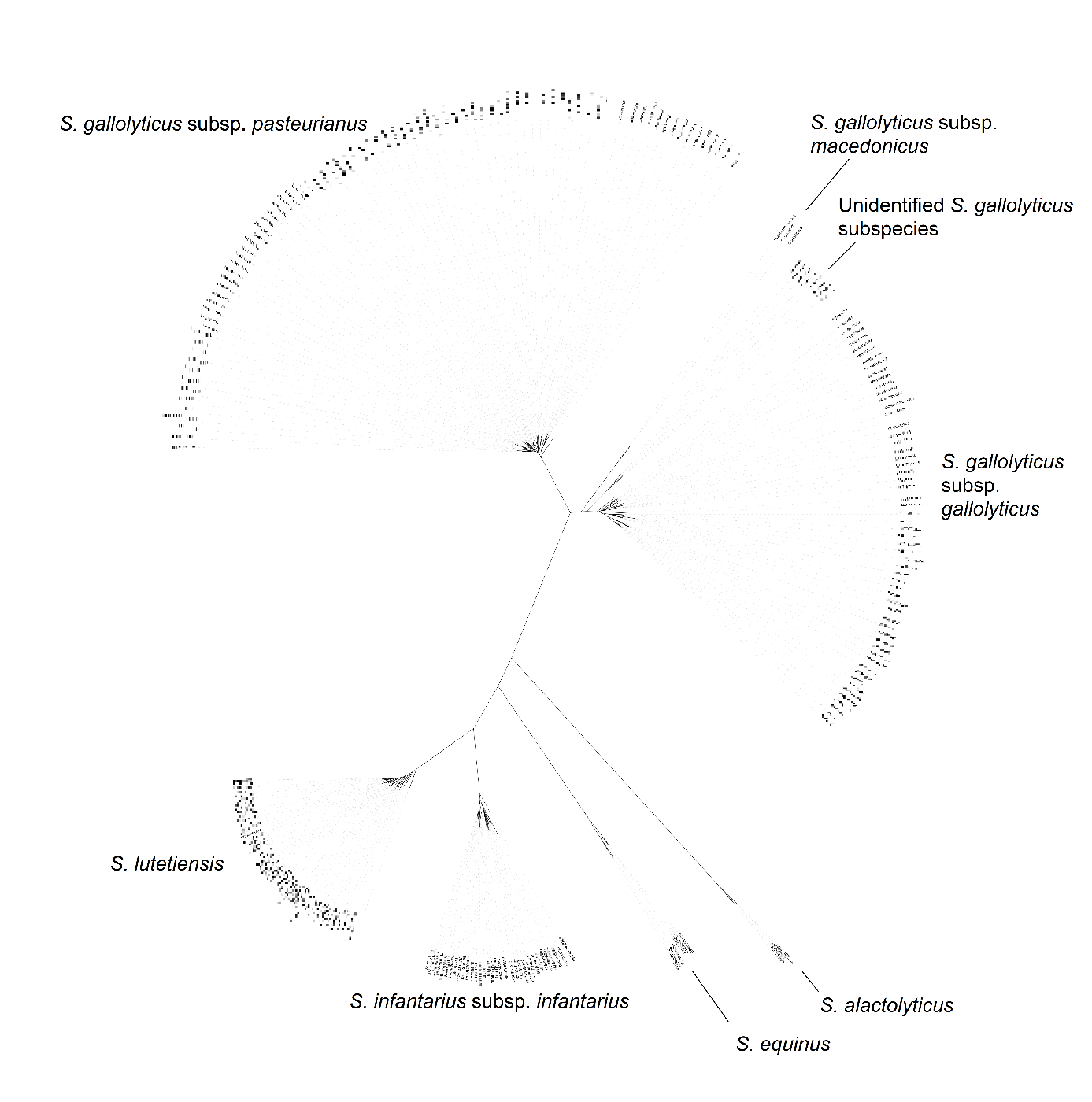
Figure S1. Clustering of subspecies in neighbor-joining phylogenetic tree.** Reference type- and library strains are included for validation purposes. Laboratory numbers are blurred due to patient confidentiality.

**Table S1. Reference type strains used.**

| **Name of type strain** | **Species** |
| --- | --- |
| CCUG 27297T | *Streptococcus alactolyticus* |
| CCUG 27302T | *Streptococcus equinus* |
| CCUG 35224T | *Streptococcus gallolyticus* subsp*. gallolyticus* |
| CCUG 39970T | *Streptococcus gallolyticus* subsp*. macedonicus* |
| CCUG 46150T | *Streptococcus gallolyticus* subsp*. pasteurianus* |
| CCUG 43820T | *Streptococcus infantarius* subsp. *infantarius* |
| CCUG 46149T | *Streptococcus lutetiensis* |

**Table S2. Incidence of SBSEC-bacteremia in the Skåne Region since introduction of MALDI-TOF MS.**

| **Year** | **SBSEC episodes** | **Population^1)^** | **Incidence Rate (per 100 000)** |
| --- | --- | --- | --- |
| 2012 | 17 | 1 263 088 | 1.35 |
| 2013 | 20 | 1 274 069 | 1.57 |
| 2014 | 26 | 1 288 908 | 2.02 |
| 2015 | 28 | 1 303 627 | 2.15 |
| 2016 | 39 | 1 324 565 | 2.94 |
| 2017 | 31 | 1 344 689 | 2.31 |
| 2018 | 27 | 1 362 164 | 1.98 |

^1)^ 31 December, Statistics Sweden [18].

**Table S3. Colonoscopy/CT colonography findings within 12 months after bacteremia ^1, 2)^.**

|  | *S. gallolyticus subsp. pasteurianus*  (n = 26) | *S. gallolyticus subsp. gallolyticus* (n = 30) | *S. lutetiensis* (n = 7) | *S. infantarius subsp. infantarius* (n = 9) | Other subspec-ies  (n = 5) | *P* |
| --- | --- | --- | --- | --- | --- | --- |
| Colorectal cancer | 2 (8) | 9 (30) | 0 (0) | 1 (11) | 0 (0) | 0.08 |
| High grade colorectal adenoma^3)^ | 0 (0) | 4 (13) | 2 (29) | 3 (33) | 0 (0) | 0.04 |
| Low-medium grade and non-biopsied colorectal adenoma and polyps^4)^ | 4 (15) | 11 (37) | 5 (71) | 5 (56) | 4 (80) | <0.01 |
| Colorectal neoplasia, total^5)^ | 6 (23) | 24 (80) | 7 (100) | 9 (100) | 4 (80) | <0.001 |

^1)^  All patients who underwent examination regardless of prior cancer diagnosis. ^2)^ Categorical variables are presented as n (% of examined). ^3)^ All were tubular-, villous-, or tubulovillous adenoma. ^4)^ Biopsy was not performed in four examinations. All other findings were of tubular-, villous-, or tubulovillous adenoma, while one was also of serrated adenoma. ^5)^ CRC, all registered adenoma or polyps in total.
